# Methacrylate Cationic Nanoparticles Activity against Different Gram-Positive Bacteria

**DOI:** 10.3390/antibiotics12030533

**Published:** 2023-03-07

**Authors:** Syong H. Nam-Cha, Ana V. Ocaña, Ramón Pérez-Tanoira, John J. Aguilera-Correa, Abraham J. Domb, Marta C. Ruiz-Grao, Sandra Cebada-Sánchez, Ángel López-Gónzalez, Milagros Molina-Alarcón, Juan Pérez-Martínez, Francisco C. Pérez-Martínez

**Affiliations:** 1 Department of Pathology, Complejo Hospitalario Universitario, 02006 Albacete, Spain; 2 Instituto de Investigación en Discapacidades Neurológicas (IDINE), University of Castilla-La Mancha, 02001 Albacete, Spain; 3 Clinical Microbiology Department, Hospital Universitario Príncipe de Asturias, 28805 Madrid, Spain; 4 Biomedicine y Biotechnology Department, School of Medicine, University of Alcalá de Henares, 28054 Madrid, Spain; 5 Clinical Microbiology Department, IIS-Fundacion Jimenez Diaz-UAM, 28040 Madrid, Spain; 6 Institute of Drug Research, School of Pharmacy-Faculty of Medicine, Center for Nanoscience and Nanotechnology and The Alex Grass Center for Drug Design and Synthesis, The Hebrew University of Jerusalem, Jerusalem 91120, Israel; 7 Department of Nursing, University of Castilla-La Mancha, 02071 Albacete, Spain; 8 Health and Social Research Center, University of Castilla-La Mancha, 16071 Cuenca, Spain; 9 BIOTYC Foundation, C/Blasco de Garay 27, 02003 Albacete, Spain; 10 Department of Nephrology, Complejo Hospitalario Universitario, 02006 Albacete, Spain

**Keywords:** DMAEMA, nanoparticles, MMA, antibacterial activity

## Abstract

Nanotechnology is a developing field that has boomed in recent years due to the multiple qualities of nanoparticles (NPs), one of which is their antimicrobial capacity. We propose that NPs anchored with 2-(dimethylamino)ethyl methacrylate (DMAEMA) have antibacterial properties and could constitute an alternative tool in this field. To this end, the antimicrobial effects of three quaternised NPs anchored with DMAEMA were studied. These NPs were later copolymerized using different methylmethacrylate (MMA) concentrations to evaluate their role in the antibacterial activity shown by NPs. Clinical strains of *Staphylococcus aureus*, *S. epidermidis*, *S. lugdunensis* and *Enterococcus faecalis* were used to assess antibacterial activity. The minimal inhibitory concentration (MIC) was determined at the different concentrations of NPs to appraise antibacterial activity. The cytotoxic effects of the NPs anchored with DMAEMA were determined in NIH_3_T_3_ mouse fibroblast cultures by MTT assays. All the employed NPs were effective against the studied bacterial strains, although increasing concentrations of the MMA added during the synthesis process diminished these effects without altering toxicity in cell cultures. To conclude, more studies with other copolymers are necessary to improve the antibacterial effects of NPs anchored with DMAEMA.

## 1. Introduction

Since antibiotics were introduced in the mid-twentieth century, they have become indispensable medicines for treating most clinical infectious processes caused by bacteria [1]. They have allowed progress in various fields of medicine, such as transplantation, prosthetic surgery, catheterization and increased survival in premature and immunosuppressed patients, not to mention that their introduction has increased the life expectancy of the population by several years. However, these drugs’ efficacy is increasingly deteriorating due to bacterial resistance to antibiotics [2]. The development of bacterial resistance along with the consequent appearance and dissemination of multidrug-resistant bacteria and scarce alternative treatments are two of the biggest problems that national health systems face today [3]. This is justified as infections by resistant bacteria are a problem that results in greater morbidity and mortality and causes economic problems. Although there are many factors that favour the selection and spread of antibiotic resistance [4], the inappropriate and indiscriminate use of antibiotics is one of the main factors contributing to this phenomenon along with a poor control of bacterial infection [5]. In Europe, according to the report of the National Plan against Antibiotic Resistance 2019–2021, it is estimated that around 33,000 people die each year from hospital infections caused by resistant germs with an annual cost of EUR 1500 million. In Spain, it is estimated that this cost is around EUR 150 million per year. It is postulated that in 35 years, the number of deaths will reach the figure of about 390,000 a year throughout Europe, and about 40,000 in Spain specifically, surpassing cancer as a cause of death [6]. At the end of the 21st century, the main health problems of antibiotic resistance in Spain were caused by Gram-positive bacteria, including methicillin-resistant *Staphylococcus aureus* (MRSA) at the hospital and macrolides- and penicillin-resistant *Streptococcus pneumoniae* at the community. Far from disappearing, these problems persist today, with a prevalence of around 25–30% for the total of both isolated pathogens. Other Gram-positive bacteria, such as glycopeptides-resistant *Enterococcus* spp., have begun to gain relevance in the last decade, but without a doubt, the greatest growing threat in our time is determined by Gram-negative bacteria, capable of accumulating resistance to available antibiotics (extensive drugs resistance or XDR), all antibiotics (pandrug-resistance, PDR) or, as with enterobacteria especially, *Pseudomonas aeruginosa* and *Acinetobacter baumannii* specifically [7].

The 2020 WHO annual report on antibacterial products under development shows a clear stagnation in their development. A small number of antibiotics have been approved in recent years, with the vast majority not being new drugs but derivatives of existing antibiotic families with already known resistance. It is therefore expected that these recently approved antibiotics will quickly create resistance. This same report also concludes that “*in general, products in clinical development and recently approved antibiotics are insufficient to address the problem posed by the increasing emergence and spread of antimicrobial resistance*” [8]. 

Applying nanotechnology in medicine and healthcare has been oriented mainly to investigating nanoparticles (NPs), nanostructures and nanodevices for the early diagnosis and treatment of neoplastic, cardiovascular, autoimmune and infectious diseases [9]. Their small sizes, which are similar to the sizes of biomolecules, confer on them a very high potential for application in medicine [10]. They are small enough to be able to interact with cell membrane receptors with high specificity and are large enough to transport drugs at the molecular level [11]. Moreover, the surface of NPs can be easily modified, which allows for greater immunocompatibility and provides a possible solution for solubility- or toxicity-related problems [12].

One of the growing fields of nanomedicine is the treatment of infectious diseases due to its antimicrobial capacity and potential use as a vector for certain antimicrobial drugs [13]. Recent studies have demonstrated their biocidal ability to eliminate *Salmonella typhi* and eradicate cancerous agents, such as *Cyanobacteria*, from the environment [14,15]. Metallic NPs have also demonstrated their antimicrobial capacity against Gram-positive bacteria (*S. aureus* and *Bacillus subtilis*) and Gram-negative bacteria (*P. aeruginosa* and *E. coli*) [16]. There are multiple examples described in the literature of such metallic NPs, for example, silver NPs with effects against Gram-negative bacteria, such as *E. coli*, *P. aeruginosa* or *Vibrio cholera*, or against Gram-positive bacteria, such as *B. subtilis*, *S. aureus* or *Enterococcus faecalis* [17,18,19]; zinc NPs that are effective against *E. coli*, *P. aeruginosa* and *S. aureus* [17]; or copper and aluminium NPs that are effective against *E. coli* and *B. subtilis* [19,20]. Other metallic NPs that appear to be useful in infections against *S. aureus* are iron NPs [21] and silica oxide NPs, which seem to be effective against *E. coli* and *B. subtilis* [20]. Thus, all these studies conclude that the antimicrobial efficacy of NPs seems to vary according to type, size, shape and concentration [13].

Polymeric NPs are biocompatible and biodegradable with excellent stability. Synthetic polymeric NPs are employed as drug delivery systems either as a polymeric drug alone or in combination with other small-molecule drugs or with biomacromolecules, such as proteins and poly (nucleic acids). Their surfaces can be modified by chemical transformations, which benefits the controllable release of different drugs [22].

Protonated polymer particles based on 2-(dimethylamino)ethyl methacrylate (DMAEMA) possess interesting properties, including antimicrobial activity against various pathogenic bacteria that may cause harmful infections [23,24]. The DMAEMA studied is a well-known polymer that responds to pH and temperature. Its weak tertiary amine groups are positively charged in solutions at a pH of around 7, while they are neutral at higher pH values [25]. DMAEMA can also be quaternised using different alkyl halides, which introduce permanent cationic quaternary ammonium salt fractions along the polymer chain. These polymer types display marked activity at temperatures below 40 °C. However, the influence of temperature can be adjusted by varying not only the degree of either the protonation or quaternization of their tertiary amine groups but also the length of the alkyl chain of the alkylating agent [25]. This type of quaternised compounds has been previously tested against Gram-positive and Gram-negative bacterial strains, both in solution and biofilm [26,27]. However, the need to fully quaternise DMAEMA units, in order to ensure effective biocidal action, remains unclear in the literature. Thus, three quaternised and copolymerized compounds were tested here using different methylmethacrylate (MMA) concentrations, and the bactericidal activity of these new compounds was successfully tested against the clinical pathogenic Gram-positive *S. epidermidis*, *S. aureus*, *S. lugdunensis* and *E. faecalis*.

## 2. Methods

### 2.1. Origin and Preparation of Nanoparticles

**Figure 1:**
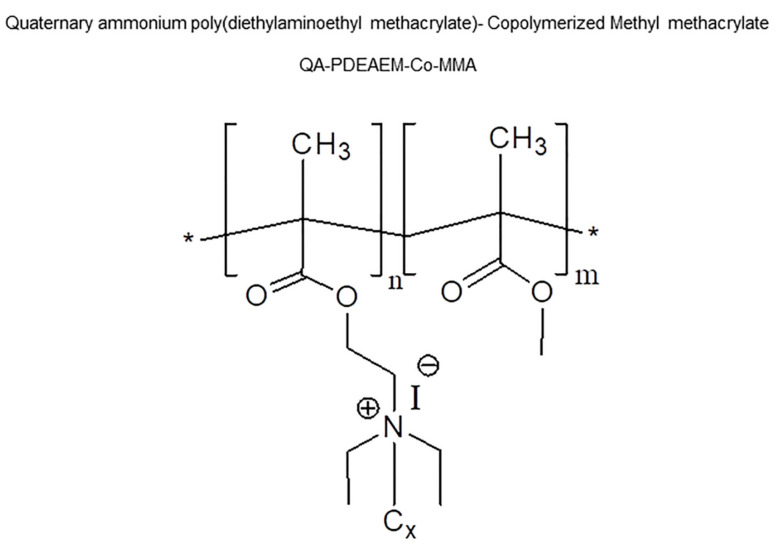
Chemical structures of the NPs anchored with DMAEMA and later copolymerized using different MMA concentrations.

NPs were prepared and characterized as previously described [26]. Briefly, the DEAEM monomer was first reacted with iodooctane to form quaternary ammonium (QA-DEAEM), which was then copolymerized with methacrylic acid using azo-bis-acrylonitrile (ABIN) as a polymerization radical source to form the three types of NPs (Figure 1). Before each cell study, NPs were thawed and resuspended in culture medium to obtain the desired working solution.

### 2.2. Cell Cultures

A commercially obtained NIH_3_T_3_ mouse fibroblast embryonic cell line (ATCC^®^ CRL-1658™, Manassas, VA, USA) was utilized. This cell line was grown in 75 cm^2^-flask culture plates with Dulbecco’s modified Eagle’s culture medium (DMEM) supplemented with 10% foetal bovine serum (FBS), according to the protocol of the commercial house ATCC (American Type Culture Collection). The culture medium was renewed every 2 days. Cell passes were performed twice weekly when they reached 80% confluence.

After obtaining enough cells, they were seeded in p24 plates with 75,000 cells per well (1.8 × 10^6^ cells for each p24 plate) for later use.

### 2.3. Toxicity Studies

Cell viability was determined by 3-(4,5-dimethylthiazole-2-yl)-2,5-diphenilltetrazolium (MTT) bromide, as described by Mosmann T. in 1983 [28], with slight modifications as previously published by our laboratory [29]. After preparing NPs with DMEM medium, cells were incubated at 37 °C at 5% CO_2_ for 24 h with the NPs at the serial concentrations of 0.2 μg/mL, 1 μg/mL, 2 μg/mL, 10 μg/mL and 20 μg/mL. After removing the incubation medium, wells were washed with PBS and cells were incubated 4 h after adding 400 μL of culture medium with MTT at a concentration of 5 mg/mL (Sigma, Barcelona, Spain).

After removing the solution, cells were resuspended in 200 μL of DMSO (Merck, Berlin, Germany) and their absorbance was determined by a microplate spectrophotometer (Asys UVM 340 microplate reader, Biochrom, Berlin, Germany) at a wavelength of 540 nm. A reference wavelength of 690 nm was also used. The cell viability results (mitochondrial activity) were expressed as a percentage of MTT transformed into formazan at all the studied concentrations of NPs in relation to the control group. As a control group, the cell line not treated with NPs was considered by taking the levels present in this group as 100%. These experiments were performed 4 times.

### 2.4. Microbiological Study Using Clinical Strains

The strains employed in this paper were the Gram-positive bacteria *S. epidermidis*, *S. aureus*, *S. lugdunensis* and *E. faecalis*. These four pathogens were clinical isolates obtained from patients with peritonitis at the Albacete University Hospital, Spain. Written informed consents were obtained from all participants. This paper was approved by the Albacete University Hospital Ethics Committee.

The frozen strains were thawed and sown on tryptic soy agar supplemented with 5% ram’s blood at 35 °C and 5% CO_2_ for at least 18 h. Next a suspension was prepared from 3–4 colonies of each bacterium, which was inoculated in a 5 mL tube with physiological serum and fluted. Finally, the obtained suspension was adjusted to a concentration on the MacFarland scale of 0.5, the equivalent to approximately 10^8^ CFU/mL.

The susceptibility of the four clinical strains to different NPs was performed by the previously described microdilution technique on a sterile round-bottomed 96-wells plate [30]. Different concentrations of the NP under study (from 0.5 to 256 μg/mL) were incubated in each well together with 50 μL of the inoculum of the corresponding bacterial strain. NPs were previously diluted in Müller–Hinton medium, according to the protocol of the commercial house ATCC. Müller–Hinton bacterial culture medium supplemented with 10% FBS was used as the negative control. The inoculum of the corresponding bacterial strain without adding NPs was utilized as the positive bacterial growth control.

To evaluate the effects of NPs on the different clinical bacterial strains, the MIC was determined after 24 h of incubation at 37 °C and 5% CO_2_. In this manner, the percentage of live bacteria was studied by taking the levels of bacteria present in the positive control as 100% and the levels of the bacteria present in the negative one as 0%. The MIC value was determined by measuring microplate absorbance at an optical density (OD) of 600 nm (Asys UVM 340 Biochrom Microplate Reader, Berlin, Germany). These experiments were performed 4 times.

Statistical tests were not used in this study. In the current study, we aimed to screen the antimicrobial activities of SC-19 to determine whether it had any potential antimicrobial effect or not. Thus, since we did not obtain quantitative results obtained from many different clinical isolates, we were unable to compare our results with a statistical method.

## 3. Results

### 3.1. Cell Viability Study by MTT Assays

**Figure 2:**
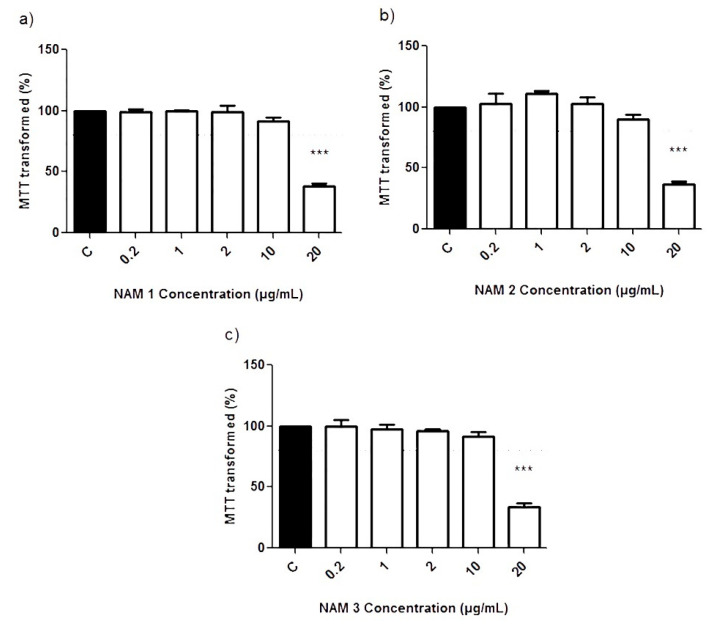
Evaluation of the cytotoxicity induced by the NPs copolymerized using DMAEMA and MMA. Cytotoxicity was determined by the percentage of MTT transformed in the NIH_3_T_3_ cells exposed to concentrations from 0.2 to 20 μg/mL of NAM1 (**a**), NAM2 (**b**) and NAM3 (**c**). Data are expressed as mean ± SEM (n = 5). *** *p* < 0.001 vs. the vehicle-treated cells (by Kruskal–Wallis, followed by Dunn’s test).

Regarding the cell viability in NIH_3_T_3_ mouse fibroblasts cultures, NAM1, NAM2 and NAM3 did not show any significant reduction in cell viability up to 10 μg/mL. In contrast, at the dose of 20 μg/mL of NAM1, NAM2 and NAM3, cell viability significantly reduced (*p* ≤ 0.05) to 38.39 ± 1.99%, 36.93 ± 2.39% and 34.17 ± 2.69%, respectively. These results indicate a similar toxicity of the three cationic NPs stabilized in DMAEMA and copolymerized with MMA (Figure 2).

### 3.2. Effects of NPs on the Gram-Positive Clinical Bacterial Strains

The results of bacterial growth obtained after the MIC experiments employing the serial dilutions of each studied NP are shown in Table 1. The doses used in each experiment ranged between 0.5 μg/mL and 256 μg/mL and were obtained by the serial dilutions of a stock concentration of 1 mg/mL.

NAM1 inhibited the bacterial growth of all the Gram-positive bacteria tested in this study in a dose-dependent manner. Thus, NAM1 inhibited *S. epidermidis* and *E. faecalis* growth at concentrations over or equalling 32 μg/mL. *S. aureus* was inhibited at concentrations above or equalling 128 μg/mL, as was *S. lugdunensis* at concentrations higher than or equalling 0.5 μg/mL.

NAM2 inhibited *S. lugdunensis* growth at concentrations above or equalling 1 μg/mL. It also inhibited the bacterial growth of *S. epidermidis* and *E. faecalis* at concentrations higher than or equalling 64 μg/mL and 32 μg/mL, respectively. NAM2 only inhibited *S. aureus* growth at concentrations that exceeded or equalled 128 μg/mL.

NAM3 inhibited the bacterial growth of all the Gram-positive bacteria used in this study. Thus, *S. epidermidis* growth was inhibited at concentrations above or equalling 64 μg/mL. The growth of both *S. lugdunensis* and *E. faecalis* was inhibited at concentrations higher than or equalling 32 μg/mL. *S. aureus* growth was inhibited at concentrations that exceeded or equalled 128 μg/mL.

## 4. Discussion

In the last few years, it has been estimated that there are more than 2.6 million people infected with multidrug-resistant bacteria per year in the USA, with a significant rise in the mortality rate related to these infections compared to previous reports [31]. Fewer antibiotics have been approved in recent years, of which the vast majority are not new drugs but derive from existing families of antibiotics with already known resistances. Thus, clinically developed products and recently approved antibiotics are insufficient to address the problem posed by the increasing emergence and spread of antimicrobial resistance [32].

With the rise of multidrug resistant microorganisms, NPs are possible future tools for use as alternative antimicrobial agents to treat these infections [33]. NPs can act by coming into direct contact with the bacterium wall without penetrating the cell and, therefore, the resistance mechanisms described in conventional antibiotics would not take place [13]. In addition, they can be easily synthesized with polymers, lipids or metals, which is an inexpensive technique and one that probably offers high clinical and economic profitability [34]. However, one of the problems that arises when using NPs in humans is toxicity and the possible side effects on the human body [35]. In line with this concern, all the NPs were used herein at lower doses than 10 μg/mL and did not show any significant changes in NIH_3_T_3_ cell viability. When analysing the results based on the synthesis of NPs, the copolymerization with MMA, performed by varying the MMA concentration used in the synthesis reaction, did not affect the toxicity produced by these NPs in eukaryotic cell cultures. Moreover, the in vitro cytotoxicity shown by the NPs employed in this study depends largely on the applied concentration and is dose-dependent toxicity. This characteristic is common to other types of NPs [36], but it is difficult to make reliable comparisons to the NPs used in other studies, because the type of study conducted to assess the toxicity of compounds and the employed cellular model play a very important role in determining the safety range of NPs [37].

Different mechanisms can explain the toxic effect of NPs, including cell membrane damage, inflammatory reactions, mitochondrial damage, reactive oxygen species (ROS) and nitric oxide synthase (NOS) production, apoptosis and necrosis [38]. Moreover, the small size of NPs and the high reactivity of nanomaterials are able to provide a greater bioavailability of NPs, which would increase their ability to be absorbed by cells of the human organism and would thus accumulate in tissues and immunological reactions or would release degradation products that are harmful to the human body [39].

DMAEMA-MMA NPs were effective in inhibiting the bacterial growth of the Gram-positive clinical strains used in the present study. These results correlated with previous studies in which the effects of cationic NPs stabilized with PDMAEMA, a polymer similar to that employed in this paper, were studied in Gram-positive strains [24]. Classically, staphylococcal species have been separated into four and eleven species groups based on a single locus with a few staphylococcal taxa [40,41,42,43,44,45]. Today, however, many phylogenetic studies based on whole-genome sequencing (WGS) are now available, which has become a powerful tool in microbiology. Lamers et al. suggested separating staphylococcal species into six major staphylococcal species groups comprising fifteen refined cluster groups. Indeed, species of heightened clinical significance, including *S. aureus*, *S. epidermidis*, *S. warneri*, *S. haemolyticus* and *S. lugdunensis*, form a well-supported clade. This supports the notion that our NPs are generally effective against this specific group of bacteria given the higher-level relation among these species [46]. Furthermore, some studies have revealed that the *S. lugdunensis* whole genome is closer to that of *S. aureus* than other coagulase-negative staphylococcal species [47].

NPs with different ratios of cationic/anionic sites were prepared by the radical copolymerization of N,N,N-octyldimethylaminoethyl methacrylate and methacrylic acid, although only NPs of 50–70 nm particle size were used in this study [27]. When analysing the results based on the synthesis of NPs, we noted that the higher the % of MMA used in synthesis, the lower the inhibitory capacity of bacterial growth in *S. epidermidis* and *S. lugdunensis* and that the antibacterial activity against *S. aureus* and *E. faecalis* was not affected. These findings suggest a limit in the potential use of the NPs as an antimicrobial agent in practical applications, as it may be difficult to achieve the desired antibacterial effects while minimizing any potential negative effects on human health or the environment. However, further studies related to the process of the synthesis of the NPs or to the study of other copolymers are required, as these could help to achieve an adequate balance between antibacterial activity and safety, thus improving the antibacterial effect of NPs.

Of all the obtained results, it is worth highlighting those of the clinical *S. lugdunensis* strains, because these was the only studied bacterial strains which, at non-toxic doses, would seem to show that NPs synthesised with low MMA concentrations (NAM1 and NAM2) had an inhibitory effect on their growth. The usefulness of these NPs seems adequate to attempt to control infections with *S. lugdunensis* because it did not present toxicity in eukaryotic cells at the doses required to inhibit the growth of this bacterium.

The positive charges of the studied NPs possibly interact with the negative net charge at the neutral pH noted in the staphylococcal cell wall. This resulted from the presence of teichoic acids, which harbour fewer positively charged D-alanine residues than negatively charged phosphate groups [48]. Indeed, previous studies have shown that cationic NPs have a bactericidal effect given the interaction of the positive charges of NPs with the negatively charged bacterial cell walls, which ultimately triggers the lysis and death of bacteria [49].

The results of this study show a possible pathway to creating new antimicrobial agents. The use of nanomaterials as antibacterial agents is acquiring great importance in the field of biomedicine, being a new possible alternative in the context of bacterial resistance, given that bacteria have a great genetic variability that allows them to change their structure [50,51]. Different steps in the biocide/bactericidal effect of NPs are described, such as absorption of NP on the bacterial surface, penetration through the bacterial wall, binding to the cytoplasmic membrane and rupture, the release of the contents to the cytoplasm and, finally, death [52,53]. It is probable that the cationic NPs of DMAEMA follow the same principles, interacting the cationic component or positive charge with the negatively charged cell surface, but these effects will be different, according to the strains studied for their different characteristics [24,52,54,55]. The advantage of cationic charge NPs being able to cross the cell membrane over anionic charge NPs has previously been described [56]. In addition, cationic DMAEMA-MMA NPs effects are related to the tested bacterial strains [24,26]. Indeed, it is known that Gram-negative bacteria are more sensitive to the bactericidal mechanism of action of NPs than Gram-positive bacteria. It would seem that the arrangement of the lipopolysaccharide and peptidoglycan layer in Gram-negative bacteria allows easier access to NPs. However, in Gram-positive bacteria, the peptidoglycan layer is thicker, which make the entry of NPs more difficult [57]. Thus, more studies are needed to understand the mechanism of interaction of the NPs of the studied quaternised DMAEMA-MMA against the different bacterial strains in an attempt to optimise their usefulness for treating the infections produced by the studied microorganisms. Not only do the physico-chemical characteristics of NPs influence their antibacterial capacity, but pH, temperature or aeration can also interfere with this capacity [58]. In addition, resistance to NPs has already been described despite using high concentrations or increasing exposure times to NPs [59], as *S. aureus* has predominantly shown in the present study. Likewise, the intrinsic mechanisms of bacteria to combat the exposure of NPs have been demonstrated, such as the secretion of proteins to induce the aggregation of NPs, the regulation of the efflux pumps of multiple drugs or the expression of ROS-sequestering enzymes [60].

Different groups have carried out similar studies using other types of NPs of different compositions, for example, quaternary ammonium or heavy metals NPs have also demonstrated the dilemma between cellular toxic effect and bactericidal or bacteriostatic dose [61]. In addition, PDMAEMA NPs have demonstrated usefulness as non-viral vectors for gene delivery [62], drug vector [63], water purification [64] or protein separation [65]. Other polymers, such as poly (2-tert-butylaminoethyl methacrylate) or PTBAEMA showed an antimicrobial effect by displacing calcium and/or magnesium particles from the bacterial membrane that leads to the disorganization of the same and its rupture [66,67].

This study has multiple limitations and further studies are necessary. First, it is essential to assess the safety of NPs anchored with DMAEMA on human cells. It is mandatory to determine the cellular toxicity and the possible effect on saprophytic and beneficial bacteria that exist in the human body [68,69,70,71,72]. These effects have not been fully characterised. The small size of NPs and the high reactivity of nanomaterials can provide greater bioavailability of NPs, increasing the ability to be absorbed by cells of the human organism, thus allowing them to accumulate in tissues, produce immunological reactions or release degradation products that are harmful to human bodies [73,74,75,76]. Second, in vivo studies are also necessary, as long as in vitro studies are promising. Third, another important issue still unresolved is research regarding the environmental impact of NPs to determine the potential capacity of NPs to accumulate in the environment and thus pose a risk to ecosystems and human health. More and more studies about the possible ecotoxicity of nanomaterials are being published, all suggesting that, despite the great advantages and potential applications of NPs, they could be harmful to ecosystems and the survival of the human species. [77,78]. In fact, research articles are already being published concerning the eco-friendly approach of the synthesis of silver NPs. Subbaiya et al. demonstrated the time- and dose-dependent cytotoxic effect against human breast cancer cell line by incubating *Streptomyces atrovirens* biomass with AgNO_3_ solution, with *S. atrovirens* being capable of producing Ag NPs by extracellular reduction [79].

Therefore, as described by other authors, cationic polymers can be considered a potential line of research on the path to achieving a new promising antimicrobial agent [24,80], although it is still unknown what the long-term effects or consequences will be. Further investigation with modifications to increase antibacterial effects and to reduce cytotoxicity is necessary.

## Figures and Tables

**Table 1:** Minimal inhibitory concentrations (MICs) of NAM1, NAM2 and NAM3 against *S. aureus*, *S. epidermidis*, *S. lugdunensis* and *E. faecalis*.

Nanoparticle	*S. aureus*	*S. epidermidis*	*S. lugdunensis*	*E. faecalis*
NAM1 (µg/mL)	128	32	0.5	32
NAM2 (µg/mL)	128	64	1	32
NAM3 (µg/mL)	128	64	32	32

## Data Availability

The data presented in this study are available on request from the corresponding author.
